# Using Baidu Index Data to Improve Chickenpox Surveillance in Yunnan, China: Infodemiology Study

**DOI:** 10.2196/44186

**Published:** 2023-05-16

**Authors:** Zhaohan Wang, Jun He, Bolin Jin, Lizhi Zhang, Chenyu Han, Meiqi Wang, Hao Wang, Shuqi An, Meifang Zhao, Qing Zhen, Shui Tiejun, Xinyao Zhang

**Affiliations:** 1 Department of Epidemiology and Biostatistics School of Public Health Jilin University Changchun China; 2 Yunnan Center for Disease Control and Prevention Yunnan China; 3 Department of Social Medicine and Health Management School of Public Health Jilin University Changchun China

**Keywords:** Baidu index, chickenpox, support vector machine regression model, disease surveillance, disease, infectious, vaccine, surveillance system, model, prevention, control, monitoring, epidemic

## Abstract

**Background:**

Chickenpox is an old but easily neglected infectious disease. Although chickenpox is preventable by vaccines, vaccine breakthroughs often occur, and the chickenpox epidemic is on the rise. Chickenpox is not included in the list of regulated communicable diseases that must be reported and controlled by public and health departments; therefore, it is crucial to rapidly identify and report varicella outbreaks during the early stages. The Baidu index (BDI) can supplement the traditional surveillance system for infectious diseases, such as brucellosis and dengue, in China. The number of reported chickenpox cases and internet search data also showed a similar trend. BDI can be a useful tool to display the outbreak of infectious diseases.

**Objective:**

This study aimed to develop an efficient disease surveillance method that uses BDI to assist in traditional surveillance.

**Methods:**

Chickenpox incidence data (weekly from January 2017 to June 2021) reported by the Yunnan Province Center for Disease Control and Prevention were obtained to evaluate the relationship between the incidence of chickenpox and BDI. We applied a support vector machine regression (SVR) model and a multiple regression prediction model with BDI to predict the incidence of chickenpox. In addition, we used the SVR model to predict the number of chickenpox cases from June 2021 to the first week of April 2022.

**Results:**

The analysis showed that there was a close correlation between the weekly number of newly diagnosed cases and the BDI. In the search terms we collected, the highest Spearman correlation coefficient was 0.747. Most BDI search terms, such as “chickenpox,” “chickenpox treatment,” “treatment of chickenpox,” “chickenpox symptoms,” and “chickenpox virus,” trend consistently. Some BDI search terms, such as “chickenpox pictures,” “symptoms of chickenpox,” “chickenpox vaccine,” and “is chickenpox vaccine necessary,” appeared earlier than the trend of “chickenpox virus.” The 2 models were compared, the SVR model performed better in all the applied measurements: fitting effect, *R*^2^=0.9108, root mean square error (RMSE)=96.2995, and mean absolute error (MAE)=73.3988; and prediction effect, *R*^2^=0.548, RMSE=189.1807, and MAE=147.5412. In addition, we applied the SVR model to predict the number of reported cases weekly in Yunnan from June 2021 to April 2022 using the same period of the BDI. The results showed that the fluctuation of the time series from July 2021 to April 2022 was similar to that of the last year and a half with no change in the level of prevention and control.

**Conclusions:**

These findings indicated that the BDI in Yunnan Province can predict the incidence of chickenpox in the same period. Thus, the BDI is a useful tool for monitoring the chickenpox epidemic and for complementing traditional monitoring systems.

## Introduction

Chickenpox is an acute infectious disease caused by the varicella-zoster virus. Children and adolescents are highly susceptible to chickenpox infection, and it is characterized by maculopapular rashes on the skin and mucous membranes with mild systemic symptoms. Chickenpox has a high incidence but low mortality rate, and it is one of the most common childhood diseases [1]. Although most cases have mild clinical symptoms, if not treated promptly, the infection may lead to postherpetic neuralgia, which affects the quality of life and can lead to death in severe cases [2].

Chickenpox is prevalent worldwide, and it has a seasonal pattern. The European region has a single peak pattern from March to May, whereas Asian countries have a double peak pattern from March to May and from December to January [3]. According to the World Health Organization, it is estimated that there are approximately 4.2 million hospitalizations and 4200 deaths due to serious complications of varicella annually worldwide, and the danger of varicella has been seriously underestimated [4]. Despite widespread immunization, varicella continues to spread and develop in many countries, such as the United States, Italy, and various European countries, due to its high transmissibility [3-6]. In China, according to an epidemiological survey of varicella in China from 2005-2019, a total of 6,442,147 cases of varicella were reported nationwide. The reported incidence of varicella increased from 41,211 cases (3.17/100,000) in 2005 to 7,979,482 cases (70.14/100,000) in 2019. The average annual incidence of chickenpox showed an increasing trend each year from 2005 to 2019, and the number of reported cases of varicella increased nearly 22 times from 2005 to 2019. Chickenpox now has one of the highest incidences in terms of preventable diseases in China [7].

Yunnan Province has a high incidence of chickenpox in China, and it ranked among the top 5 provinces in the country in 2019 in terms of the incidence in the population aged under 14 years, accounting for 76.36% of all chickenpox cases. Chickenpox public health emergencies mainly occur in rural elementary schools, and the situation is serious as the chickenpox epidemic continues to grow year by year. However, attention to the management and elimination of chickenpox public health emergencies in rural elementary schools and kindergartens is still insufficient, and the problem of untimely reporting of chickenpox incidence remains. Yunnan Province needs to take effective measures to suppress the spread and prevalence of chickenpox [8].

Currently, problems such as low chickenpox vaccine coverage, the breakthrough of the chickenpox vaccine, the lack of attention to chickenpox disease, imperfect chickenpox surveillance reports, and the lack of public information on chickenpox epidemics are still present in China [9]. In China, chickenpox is reported through the Chinese Disease Prevention and Control Information System. Because chickenpox is a viral disease with mild and self-limiting symptoms, it has not been included in the Chinese National Disease Reporting System for statutory infectious diseases. There is no uniform standard for reporting chickenpox cases across China. However, the public health emergency management information system has focused on some aggregated outbreaks [9,10], but some disseminated cases or subclinical infections may be overlooked.

At present, the means of chickenpox prevention and control in China are relatively singular and limited to the traditional reported incidence monitoring and isolation treatment of patients with chickenpox [2]. The actual incidence of chickenpox in most provinces is not publicly available, and the public cannot obtain timely information on the chickenpox epidemic. Apart from public health departments, the public does not have effective channels to obtain real-time information on local chickenpox incidence, which is not conducive to taking active self-protective measures against chickenpox.

The internet has become an increasingly popular means of accessing health information. The increase in web-based information provides a potentially useful source of data for disease surveillance. Due to its real-time nature and ease of access, internet data can be used to fill the gaps in traditional public health surveillance [11]. Therefore, the possibility of using internet data for the surveillance of various diseases is being increasingly explored as an aid to improve disease prevention and control. The use of internet search data as a complementary means to traditional infectious disease surveillance methods is particularly applicable to neglected diseases that are less affected by consultations [12].

Previous studies [12,13] have indicated that Google Trends can well demonstrate the epidemic characteristics of chickenpox abroad and is suitable for the simulation of features of infectious diseases in epidemic cycles. Moreover, Bakker et al [12] studied chickenpox incidence patterns using Google Trends and found that Google search data show a high correlation (*R*^2^=0.65-0.71) with varicella outbreaks. Thus, Google Trends can be used for chickenpox incidence prediction and early warning. In China, more studies have focused on vaccine efficacy analysis and descriptive studies of chickenpox prevalence [7-10], and most of the exploration of chickenpox surveillance issues has been performed by using actual incidence report data to build models to predict the onset of chickenpox [2], whereas there are few studies exploring whether the Baidu search engine can be applied to chickenpox epidemic surveillance and early warning.

Google Trends is not highly used in China. Google search data do not reflect the true search tendencies of the Chinese public because Baidu is the most widely used search engine product in China. The Baidu index (BDI) [14], which was established based on Baidu search information, should reflect the search needs and awareness of internet users as well as Google Trends abroad in China [15,16]. A considerable number of studies have been conducted using the BDI for China-wide disease prediction. For example, one study has successfully predicted the epidemic trend of influenza using the BDI [17]. Similarly, some surveillance studies using BDIs for diseases, such as brucellosis; dengue fever; and hand, foot, and mouth disease, have shown that BDIs can be used to reflect disease prevalence [18-20].

The current chickenpox surveillance system in China is flawed and incomplete. The flawed surveillance system and the lack of public information on chickenpox outbreaks have led to a weak response to chickenpox outbreaks in China. Public health events caused by chickenpox epidemics have persisted for a long time, generating a large number of patients with chickenpox, which disrupts the study, work, and life of patients while also affecting the normal production and everyday life of families, schools, and workplaces. Moreover, chickenpox epidemics impose a heavy burden on the healthy socioeconomic development of China.

This study focused on the following specific research questions:

1. What are the correlations between BDI scores and actual varicella incidence data in Yunnan Province?
2. Can internet data be used to predict future varicella disease epidemics?
3. Can big data be used as a supplement to traditional surveillance systems for early warning surveillance of infectious diseases as well as epidemics?

## Methods

### Real-World Databases

The data were divided into 2 parts as follows: the number of reported chickenpox cases in Yunnan Province from 2017 to 2021 and the chickenpox-related BDI search data in Yunnan Province from 2017 to April 2022. The chickenpox monitoring data in Yunnan Province were obtained from the chickenpox epidemic information of the Yunnan Province Center for Disease Control and Prevention.

### BDI Databases

The chickenpox-related BDI search data were obtained from the official website of the BDI [14]. The BDI of “PC + mobile” from 2017 to April 2022 was collated as search data.

The principles of selecting keywords were as follows: (1) keywords must be closely related and specific to chickenpox; (2) selected longtail keywords were considered for inclusion; (3) keywords must have sufficient search volume in the mining module; and (4) the time series of each keyword must be complete and valid.

The following 12 keywords were selected: “chickenpox,” “chickenpox pictures,” “chickenpox symptoms,” “chickenpox diet,” “chickenpox infection period,” “symptoms of chickenpox,” “symptoms and treatment of chickenpox,” “chickenpox vaccine,” “is chickenpox vaccine necessary,” “shingles,” “chickenpox treatment,” and “treatment of chickenpox.”

### Analysis

#### Keyword Screening and Relevance Test

First, Pearson and Spearman correlation tests were performed on the first 12 selected BDIs and the number of chickenpox cases, with Pearson correlation coefficient>0.3 as the criteria for determining the correlation. A time-lagged cross-correlation test was then performed on the screened keyword indices and the number of chickenpox cases to determine the time type of keywords (prior, simultaneous, and lagged).

#### Support Vector Machine Regression Model Construction

For variable selection, the number of chickenpox cases in Yunnan Province from January 2017 to May 2021 was used as the dependent variable, and the weekly BDIs of 8 keywords, such as “chickenpox,” were used as the independent variables. The BDIs and incidence data of 209 weeks from 2017 to 2020 were used as the training set, and the data of 22 weeks from January to May 2021 were used as the test set. The data were fitted with the actual incidence data to analyze the accuracy.

#### Multiple Linear Regression Model Construction

The general expression was as follows:

y = β_0_ + β_1_x_1_ + β_2_x_2_ +...+ β_k_x_k_ + ε

where *β_0_, β_1_, β_2_, ..., β_k_* are the parameters of the model, and ε is the error term.

The variable selection was consistent with the support vector machine regression (SVR) model. For model construction, the stepwise regression method was used to eliminate the independent variables that had no significant effect (*P*>.05) on the dependent variable, and the model with the highest *R*^2^ value of goodness-of-fit was selected for the next step of prediction according to the stepwise regression model construction results.

#### Determination Index of the Optimal Model

The optimal model for the present study was selected by comparing the coefficient of determination (*R*^2^), the mean absolute error (MAE), and the root mean square error (RMSE) of the predicted and actual values of the above 2 models in fitting and predicting data.

#### Data Prediction of the Chickenpox Epidemic in Yunnan Province

The optimal prediction model was selected by the above method. The BDI of the “chickenpox” keyword and the number of reported chickenpox cases in Yunnan Province for 231 weeks from January 2017 to May 2021 were imported as independent and dependent variables to establish the prediction model. The BDI data from June 2021 to the first week of April 2022 were obtained to predict the trend of chickenpox incidence in Yunnan Province.

### Ethical Considerations

The data sources for this study included publicly available BDI search data and varicella incidence data provided by the Yunnan Province Center for Disease Control and Prevention. As this study did not involve human or animal experimental subjects, nor did it involve any ethical issues related to data collection or use, ethical approval or a license was not required for this study.

## Results

### Cross-Correlation Analysis of Chickenpox and the BDI in Yunnan Province

After the cross-correlation test, the results showed that most BDI search terms, such as “chickenpox,” “chickenpox treatment,” “treatment of chickenpox,” “chickenpox symptoms,” and “chickenpox virus,” trend consistently. Some BDI search terms, such as “chickenpox pictures,” “symptoms of chickenpox,” “chickenpox vaccine,” and “is chickenpox vaccine necessary” appeared earlier than the trend of “chickenpox virus.” In all, 8 keywords were screened from the 12 chickenpox-related keywords as variables required for modeling: “chickenpox picture,” “chickenpox,” “chickenpox symptoms,” “symptoms of chickenpox,” “symptoms and treatment of chickenpox,” “chickenpox vaccine,” “treatment of chickenpox,” and “chickenpox treatment” (Table 1).

**Table 1:** Cross-correlation analysis of actual chickenpox cases and internet search terms from Yunnan, China^a^.

Search terms			Lag (weeks)						
		–3		–2	–1	0	1	2	3
**水痘** **图** **片** **(chickenpox picture)**									
	*r^b^*	0.702		*0.730^c^*	0.715	0.705	0.636	0.555	0.462
	*P* value^d^	<.001		<.001	<.001	<.001	<.001	<.001	<.001
**水痘** **(chickenpox)**									
	*r*	0.656		0.718	0.727	*0.747^c^*	0.741	0.678	0.609
	*P* value	<.001		<.001	<.001	<.001	<.001	<.001	<.001
**水痘症状** **(chickenpox symptoms)**									
	*r*	0.364		0.37	0.371	*0.372^c^*	0.331	0.287	0.237
	*P* value	<.001		<.001	<.001	<.001	<.001	<.001	<.001
**水痘的症状** **(symptoms of chickenpox)**									
	*r*	0.550		*0.578^c^*	0.548	0.406	0.472	0.390	0.298
	*P* value	<.001		<.001	<.001	<.001	<.001	<.001	<.001
**水痘的症状和治** **疗** **(symptoms and treatment of chickenpox)**									
	*r*	0.302		0.351	0.379	*0.556^c^*	0.385	0.345	0.320
	*P* value	<.001		<.001	<.001	<.001	<.001	<.001	<.001
**水痘疫苗** **(chickenpox vaccine)**									
	*r*	*0.353^c^*		0.345	0.317	0.317	0.282	0.215	0.150
	*P* value	<.001		.001	<.001	<.001	<.001	.001	.02
**水痘的治** **疗** **(treatment of chickenpox)**									
	*r*	0.259		0.261	0.308	*0.323^c^*	0.264	0.267	0.199
	*P* value	<.001		<.001	<.001	<.001	<.001	<.001	.002
**水痘治** **疗** **(chickenpox treatment)**									
	*r*	0.216		0.272	0.294	*0.325^c^*	0.293	0.283	0.278
	*P* value	.001		<.001	<.001	<.001	<.001	<.001	<.001
**水痘** **饮** **食** **(chickenpox diet)**									
	*r*	0.194		0.196	0.187	*0.222^c^*	0.157	0.180	0.170
	*P* value	.003		.003	.004	.001	.02	.006	.01
**水痘** **传** **染期** **(chickenpox transmission period)**									
	*r*	0.165		0.200	0.219	*0.280^c^*	0.275	0.292	0.295
	*P* value	.01		.002	.001	<.001	<.001	<.001	<.001
**水痘疫苗有必要打** **吗** **(is chickenpox vaccine necessary)**									
	*r*	0.264		*0.200^c^*	0.144	0.090	–0.001	–0.078	–0.161
	*P* value	<.001		.002	.03	.17	.99	.24	.02
**带** **状疱疹** **(herpes zoster)**									
	*r*	0.024		*0.022^c^*	0.008	0.011	0.000	–0.010	–0.017
	*P* value	.72		.74	.91	.87	>.99	.88	.79

^a^Spearman correlation coefficient>0.3 was used as the criteria for inclusion.

^b^*r* values represented cross correlation coefficient.

^c^Italicized values showed the maximum of cross correlation coefficient.

^d^*P* values represented statistical significance between 2 variables.

### Time Series Characteristics and Correlation Analysis of Varicella and BDI in Yunnan Province

The number of reported chickenpox cases in Yunnan Province showed an increasing trend year by year, especially during the winter chickenpox susceptibility period in 2020, with a peak higher than the general level in previous years. The trend of chickenpox incidence in 2017-2021 showed seasonality with double peaks in the number of cases from May to July and from November to January in each year. The BDI data for the same period also showed bimodal peaks from May to July and from November to January in each year. Comparison charts of the trend of 8 BDI keywords and actual occurrence are shown in Multimedia Appendix 1.

### Construction and Prediction of Chickenpox Case Prediction Model

#### Establishment of the SVR Prediction Model

In the SVR model, the radial basis kernel function was used. We chose all 8 key terms for modeling: “chickenpox picture,” “chickenpox,” “chickenpox symptoms,” “symptoms of chickenpox,” “symptoms and treatment of chickenpox,” “chickenpox vaccine,” “treatment of chickenpox,” and “chickenpox treatment.” Using the grid search method for the hyperparameter search, the final obtained parameters were C=1 and γ=0.1.

**Figure 1:**
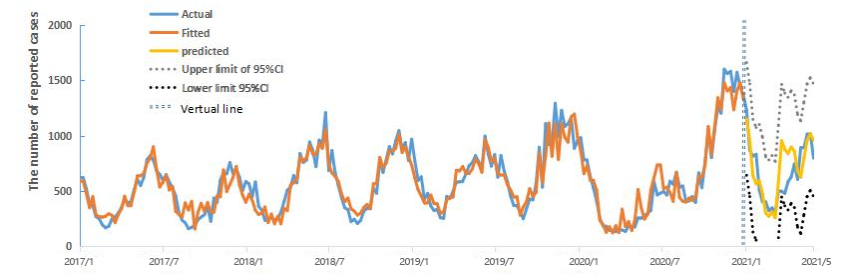
Predicted effect of support vector machine regression fitting for chickenpox in Yunnan Province (January 2017 to May 2021).

The complete fitting and prediction effect graph is shown in Figure 1.

#### Multiple Linear Regression Prediction Modeling

**Figure 2:**
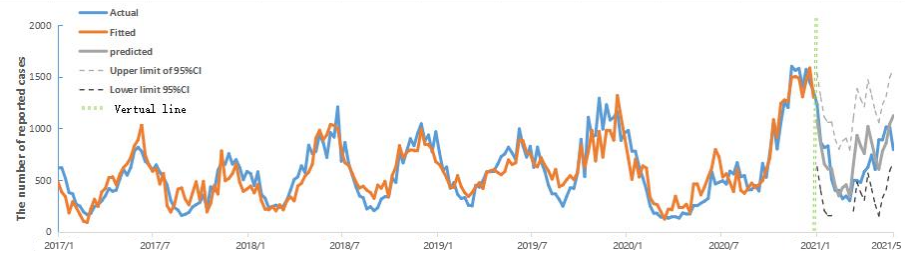
Predicted effect of multiple linear regression fitting for chickenpox in Yunnan Province (January 2017 to May 2018).

In the multiple linear regression model, we also chose all 8 key terms for modeling. We used the stepwise regression method to fit and test the data. The model performed best when those 4 key terms were included, namely “chickenpox,” “chickenpox symptoms,” “symptoms of chickenpox,” and “treatment of chickenpox.” The detailed regression coefficients are as follows: b_1_=0.243, b_2_=0.495, b_3_=0.381, and b_4_=0.278. The complete fitting and prediction effect graph is shown in Figure 2.

#### Comparison of Multiple Regression Model and SVR Model Prediction Performance

To evaluate the fitting performance of the model, we used *R*^2^, MAE, and RMSE to compare the advantages and disadvantages of the 2 models. The results showed that the SVR model had better performance in both fitting and prediction effects compared to the multiple linear regression model (Tables 2 and 3).

Therefore, SVR was selected as the best model for case number prediction.

**Table 2:** Comparison of model fitting effect indicators.

	MLR^a^ model	SVR^b^ model
*R* ^2^	0.833	0.911
RMSE^c^	130.3389	96.2995
MAE^d^	106.6526	73.3988

^a^MLR: multiple linear regression.

^b^SVR: support vector machine regression.

^c^RMSE: root mean square error.

^d^MAE: mean absolute error.

**Table 3:** Comparison of model prediction effect indicators.

	MLR^a^ model	SVR^b^ model
*R* ^2^	0.459	0.548
RMSE^c^	204.2203	189.1807
MAE^d^	166.2412	147.5412
MAPE^e^	15%	9.1%

^a^MLR: multiple linear regression.

^b^SVR: support vector machine regression.

^c^RMSE: root mean square error.

^d^MAE: mean absolute error.

^e^MAPE: mean absolute percentage error.

#### SVR–Based Prediction of the Number of Chickenpox Cases

**Figure 3:**
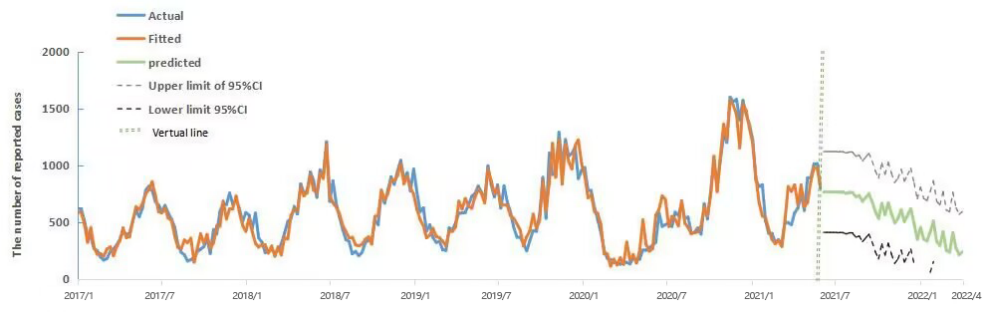
Fitting effect and prediction results of chickenpox in Yunnan Province based on the support vector machine regression model.

SVR prediction was performed using the BDI with the number of cases from January 2018 to May 2021 to predict the likely number of chickenpox cases from June 2021 to the first week of April 2022. The predicted trends are shown in Figure 3 and Multimedia Appendix 2.

## Discussion

### Principal Findings

In this study, we used the chickenpox BDI to build a model to predict the incidence of chickenpox during the same period. We concluded that it is feasible to use BDI data for varicella epidemic surveillance, which does not rely on actual reported data of varicella incidence.

### The Principle of Using the BDI to Predict Incidence

The BDI reflects changes in public interest in various events over a period of time. As a relatively neglected disease, news reports about chickenpox are substantially fewer than those about other infectious diseases, such as influenza and AIDS. Possible reasons for public interest in chickenpox are news reports or illnesses in their own family, suggesting that the main source of web-based search data may come from patients with chickenpox and their family members. This hypothesis is the reason why we believe that the chickenpox BDI can reflect the dynamics of chickenpox incidence through search information.

The use of internet data for disease surveillance has several advantages. For instance, it is more cost-effective and less time-consuming compared to traditional surveillance methods that rely on patient consultations and medical records [21,22]. Moreover, internet data can capture disease trends earlier than traditional surveillance methods because they are updated in real time. In addition, they can detect disease outbreaks that may go unnoticed using traditional surveillance methods. As neglected diseases often go unnoticed due to the limited health care resources and infrastructure in low-income countries, internet data can serve as a useful tool for monitoring these diseases. By leveraging internet data, health officials can detect disease outbreaks earlier, allocate resources more effectively, and ultimately prevent the spread of diseases more efficiently. Therefore, the use of internet data for disease surveillance has the potential to revolutionize disease prevention and control efforts.

Based on this principle, we believe that the BDI can be used as a new infectious disease surveillance tool to complement traditional public health surveillance systems. By combining big data epidemiological surveillance methods with traditional epidemiological surveillance tools, governments can establish a more sensitive surveillance tool. Real-time and low-cost internet data can help government health departments quickly identify diseases, populations, and areas with potential transmission risks and take effective measures.

### Comparison With Prior Work

This study explores the potential of using internet data for disease surveillance, with a focus on chickenpox. Although previous research has shown success in using Google for disease prediction [21-23], we wanted to investigate whether Baidu, a search engine with higher usage rates in China, could also be used for this purpose. Our findings confirm that Baidu can be used for disease monitoring, providing an opportunity to better leverage internet data to predict disease occurrence and its spread. This discovery is particularly important for public health institutions, which can now make more accurate predictions and take more targeted preventive measures. Furthermore, this study highlights the growing importance of internet information as a source of data for disease surveillance in the future.

In this study, the chickenpox BDI was highly consistent with the actual occurrence trend of chickenpox (*r*=0.747), and compared to the disease studies conducted using the BDI [15,17-20,24-27], the correlation coefficients of these studies were generally between *r*=0.3 and *r*=0.93, indicating that the chickenpox BDI well reflects the actual trend of chickenpox occurrence. Thus, it is feasible to use the BDI for chickenpox epidemic surveillance. At the same time, studies exploring the correlation between Google Trends and chickenpox [12] have shown that the average correlation between chickenpox and Google Trends is approximately *r*=0.762 globally, indicating that the use of the BDI for chickenpox in China has similar surveillance effects as those obtained using Google Trends abroad. Therefore, in China, the methods and ideas of using Google for varicella incidence trend prediction in foreign countries can be applied to the BDI to conduct supplementary surveillance of chickenpox incidence.

In this study, the SVR model outperformed the multiple linear regression model both in terms of fitting and prediction (Tables 2 and 3), which showed a mean absolute percentage error of 9.1%, RMSE of 189, and *R*^2^ of 0.548. Comparing it with previous studies [19,28], the SVR model outperformed the autoregressive model for sexually transmitted diseases and the autoregressive integrated moving average model for brucellosis in terms of mean absolute percentage error and RMSE. Overall, the SVR model showed promising results for predicting chickenpox incidence and can be further improved and compared with other models for infectious diseases in future studies.

The SVR model developed using the BDI accurately predicted the actual number of current varicella cases. Unlike previous chickenpox prediction models, the method developed in this study provided a rapid assessment of the current chickenpox epidemic without relying on actual incidence reporting data. The method allows for immediate calculation of chickenpox case prediction, which is more rapid than traditional prediction systems, allowing it to be used as a rapid method to help the public know the current chickenpox dynamics. Moreover, due to the simplicity of the method and easy access to data sources, it is likely to be applicable to most places in China. When model predictions increase, it may indicate a rise in the number of chickenpox cases, allowing for disease control and prevention–related authorities to prepare for potential chickenpox outbreaks.

### Limitations

This study had several limitations that deserve further discussion. First, China is a vast country with regional differences in customs and culture as well as population distribution and geographic ethnicity [29]. Thus, it is not possible to predict the incidence trend in all provinces using one model. If data can be collected for each region, the prediction of chickenpox incidence trends can be tailored to different regions. Second, similar to other studies exploring the relationship between disease and the internet [23], when the media reports the chickenpox epidemic, people without chickenpox may search for chickenpox out of curiosity and fear, which would lead to a surge in the number of searches. Thus, further research is needed to eliminate the influence of the media on the results. Finally, multiple regression modeling based on BDI data alone does not allow for accurate prediction of chickenpox dynamics, and the volume of disease search data does not correspond one to one with the number of reported cases. Infectious disease prediction models based solely on internet search data may also be confounded by searchers’ knowledge of the disease and local language restrictions.

Therefore, in future studies, we should also consider additional influencing factors related to chickenpox outbreaks, such as climate, economy, and vaccination status, for a comprehensive analysis to achieve accurate prediction of chickenpox incidence.

### Conclusions

Based on the results, it is feasible to apply the BDI method to reflect the incidence of varicella. The fitted and predicted values of the SVR model were consistent with the actual incidence trend of chickenpox, indicating that the model based on the BDI can be used to reflect the actual local incidence trend of chickenpox in real time. Thus, the BDI can be used for disease surveillance. Internet search data can be used as a supplement to traditional surveillance systems to help with the early detection of potential disease outbreaks or disease epidemics.

## Appendix

Multimedia Appendix 1 Comparison charts of trends of 8 additional Baidu index keywords and actual occurrences.

Multimedia Appendix 2 Support vector machine regression model prediction results.

## Abbreviations

BDI: Baidu index
MAE: mean absolute error
RMSE: root mean square error
SVR: support vector machine regression model

## Data Availability

The data sets generated during and analyzed during the current study are available from the corresponding author on reasonable request.
