# Supplementary material for: Using Baidu Index Data to Improve Chickenpox Surveillance in Yunnan, China: Infodemiology Study
Source: J Med Internet Res. 2023 May 16;25:e44186. doi: 10.2196/44186 (PMC10230353; doi:10.2196/44186)

**Multimedia Appendix 1. Comparison Chart of Trends in Seven Additional Baidu Index Keywords and Actual Occurrences**


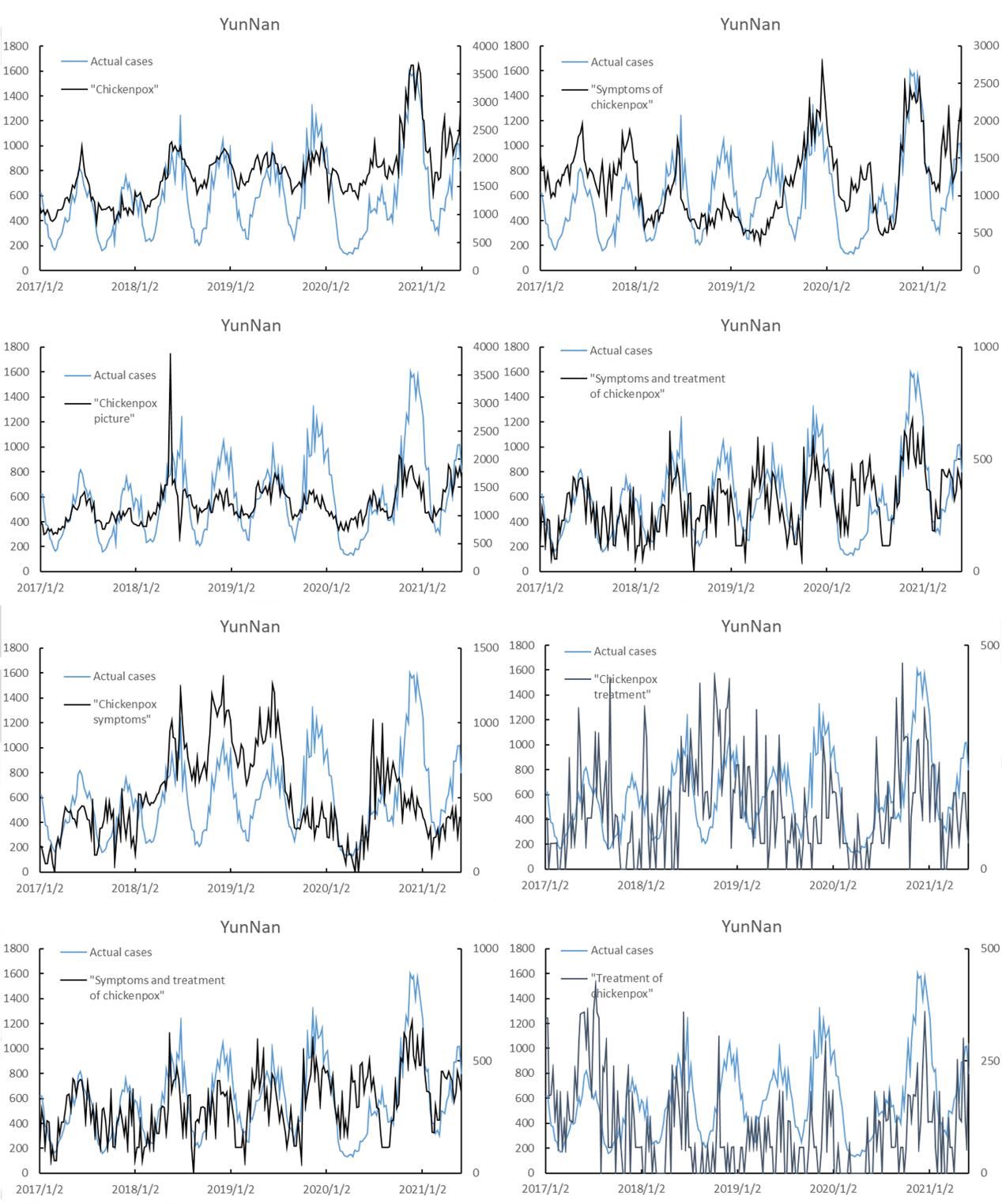

Supplement: Multimedia Appendix 1 [file jmir_v25i1e44186_app1.docx]
