# Supplementary material for: Using Baidu Index Data to Improve Chickenpox Surveillance in Yunnan, China: Infodemiology Study
Source: J Med Internet Res. 2023 May 16;25:e44186. doi: 10.2196/44186 (PMC10230353; doi:10.2196/44186)
Supplement: Multimedia Appendix 2 [file jmir_v25i1e44186_app2.docx]

**Table S1.** Support vector machine regression model prediction results.

| The first day of the week | Prediction (chickenpox cases), n |
| --- | --- |
| June 6, 2021 | 766.72186 |
| June 14, 2021 | 766.71023 |
| June 21, 2021 | 766.72028 |
| June 28, 2021 | 766.60844 |
| July 7, 2021 | 764.47741 |
| July 12, 2021 | 766.15064 |
| July 19, 2021 | 763.21337 |
| July 26, 2021 | 753.91591 |
| August 2, 2021 | 763.07149 |
| August 9, 2021 | 756.41887 |
| August 16, 2021 | 725.0648 |
| August 23, 2021 | 734.67123 |
| August 30, 2021 | 684.98862 |
| September 6, 2021 | 717.52025 |
| September 13, 2021 | 751.58736 |
| September 20, 2021 | 683.56196 |
| September 27, 2021 | 595.54058 |
| October 4, 2021 | 533.799 |
| October 11, 2021 | 666.66037 |
| October 18, 2021 | 567.58738 |
| October 25, 2021 | 671.6693 |
| November 1, 2021 | 575.54032 |
| November 8, 2021 | 494.83322 |
| November 15, 2021 | 532.44904 |
| November 22, 2021 | 609.79723 |
| November 29, 2021 | 506.57788 |
| December 6, 2021 | 563.15041 |
| December 13, 2021 | 619.32083 |
| December 20, 2021 | 489.06691 |
| December 27, 2021 | 351.14203 |
| January 3, 2022 | 454.79513 |
| January 10, 2022 | 351.20313 |
| January 17, 2022 | 337.17268 |
| January 24, 2022 | 415.8057 |
| January 31, 2022 | 509.58851 |
| February 7, 2022 | 328.92751 |
| February 14, 2022 | 297.33693 |
| February 21, 2022 | 416.76898 |
| February 28, 2022 | 251.1225 |
| March 7, 2022 | 237.02256 |
| March 14, 2022 | 407.19243 |
| March 21, 2022 | 272.10036 |
| March 28, 2022 | 214.14784 |
| April 4, 2022 | 241.31517 |
